# Concerted action of the PHD, chromo and motor domains regulates the human chromatin remodelling ATPase CHD4

**DOI:** 10.1016/j.febslet.2012.06.017

**Published:** 2012-07-30

**Authors:** Rosa Morra, Benjamin M. Lee, Heather Shaw, Roman Tuma, Erika J. Mancini

**Affiliations:** a Division of Structural Biology, Wellcome Trust Centre for Human Genetics, Oxford University, Roosevelt Drive, Oxford OX3 7BN, United Kingdom; b Biochemistry Department, Oxford University, South Parks Road, Oxford OX1 3QU, United Kingdom; c Ludwig Institute for Cancer Research Ltd., Oxford University, Old Road Campus Research Building, Headington, Oxford OX3 7DQ, United Kingdom; d Astbury Centre for Structural Molecular Biology and Institute of Cellular and Molecular Biology, University of Leeds, LS2 9JT, United Kingdom

**Keywords:** Chromatin remodelling, Histones binding, Nucleosomes, Chromodomain, PHD, ATPase, CHD4, NURD

## Abstract

CHD4, the core subunit of the Nucleosome Remodelling and Deacetylase (NuRD) complex, is a chromatin remodelling ATPase that, in addition to a helicase domain, harbors tandem plant homeo finger and chromo domains. By using a panel of domain constructs we dissect their roles and demonstrate that DNA binding, histone binding and ATPase activities are allosterically regulated. Molecular shape reconstruction from small-angle X-ray scattering reveals extensive domain-domain interactions, which provide a structural explanation for the regulation of CHD4 activities by intramolecular domain communication. Our results demonstrate functional interdependency between domains within a chromatin remodeller.

## Highlights

► We dissect the roles of the individual domains of CHD4. ► The chromo domains bind DNA while the PHD domains modulate this activity. ► ATPase and chromo domains increase the histone tail binding affinity of PHD domains. ► PHD and chromo domains are required for DNA stimulated ATPase activity. ► SAXS shape reconstruction delineates intramolecular interactions between domains.

## 1 Introduction

Chromodomain helicase DNA binding protein 4 (CHD4), also known as Mi2β, belongs to the SNF2 family of helicases [1] and was first identified as a dermatomyositis-specific autoantigen [2]. CHD4 is the main subunit of the Nucleosome Remodelling and Deacetylase (Mi-2/NuRD) complex, a chromatin remodelling complex which is involved in many fundamental biological processes [3,4]. Mi-2/NuRD is thought to act as transcriptional repressor working in opposition to other chromatin remodellers such as SWI/SNF [5]. Like other chromatin remodelling complexes, Mi-2/NuRD achieves diversity in regulatory function through combinatorial assortment of its motor protein, CHD4, with other subunits including histone deacetylases HDAC1 and HDAC2 [6]. Despite the wealth of information available for CHD4, little is known on how this ATPase is targeted to specific sites within a chromatin environment and about the molecular mechanism of its chromatin remodelling activity.

In addition to its SNF2-type ATPase domain, the 218 kDa CHD4 protein harbours tandem plant zinc finger homeodomains (tPHD), which are found in a number of chromatin remodelling factors involved in nucleosome/histone binding [7–9]*,* and tandem chromodomains (tCHD) which have been shown to mediate chromatin interaction by binding directly to either DNA, RNA or methylated histone H3 [10–13]. While the combination of tPHD and tCHD is a characteristic specific to CHD4 and two other members of the CHD family (CHD3 and CHD5) [14], the simultaneous presence of several histone-binding modules is a characteristic of many chromatin remodelling ATPases. So far, the mechanism by which these domains cooperate, and their role in the context of regulation of the ATPase motor and nucleosome remodelling remains unclear.

Here we dissect the roles of the individual domains of CHD4 and investigate their contribution to its enzymatic activity and targeting specificity. Low-resolution shape reconstructions, obtained from small angle X-ray scattering, suggest that the tight cooperation between the domains is mediated by intramolecular interactions due to their spatial proximity within CHD4. We present a plausible regulatory mechanism necessary for the nucleosome remodelling activity of CHD4.

## 2 Materials and methods

### 2.1 Expression and purification of CHD4 constructs

C-terminal 8xHis-tagged constructs were generated by PCR using human CHD4 cDNA (Mammalian Gene Collection) as a template and appropriate sets of primers. The amplified PCR products were transferred into the expression pTriEx2 (Novagen) vector, verified by DNA sequencing, and used to transform *Escherichia coli* BL21 (DE3) cells. Expression in LB or TB media was induced with 0.7 mM of isopropyl-thiogalactopyranoside (IPTG) when culture reached OD_600_ = 0.6 and incubated overnight at 20 °C. Cells were lysed in 50 mM sodium phosphate pH 7.5, 500 mM NaCl, 5 mM imidazole, 0.2% Tween 20, protease inhibitor cocktail tablets (Roche) and the obtained supernatant mixed with Talon^TM^ resin (Clontech). The recombinant proteins were eluted with imidazole gradients and further purified by size exclusion chromatography in 20 mM Tris pH 7.5, 200 mM NaCl, 1 mM DTT.

### 2.2 MALS

MALS experiments were carried out using an analytical Superdex S200 or S75 10/30 column (GE Healthcare) with online static light scattering (DAWN HELEOS II, Wyatt Technology, Santa Barbara, CA), differential refractive index (Optilab rEX, Wyatt Technology) and Agilent 1200 UV (Agilent Technologies) detectors.

### 2.3 EMSA

DNA binding and its dependence on the probe length was simultaneously probed using molecular weight markers (Gene Ruler 100 bp Plus DNA ladder, Fermentas; MW marker XIII 50–750, Roche; MW marker Gene Ruler 1 Kb and 100 bp Plus DNA ladder, Invitrogen) while 500 nt long ssRNA transcript was used for RNA. Probes were mixed with increasing amounts of CHD4 constructs in 20 μl of binding buffer (20 mM Tris pH 7.5, 200 mM NaCl), incubated for 5 min at room temperature and then run on a 2% agarose gel. In the case of the titration of tPHD into tCHD, the proteins were mixed in 20 mM Tris pH8, 200 mM NaCl buffer and incubated at RT for 30 min.

### 2.4 Nucleosome core particle mobility shift assay

Isolated radiolabeled mononucleosomes (50 fmol) reconstituted by salt gradient dialysis from recombinant *X. laevis* histones and a 168 bp fragment of 601-DNA (as described in [15] were incubated with increasing amount of CHD4 constructs for 15 min on ice in 10 μl of binding buffer (20 Tris–HCl pH 8, 200 mM NaCl, 10% sucrose). To control for nucleosome dissociation, any DNA released from the nucleosomes was chased with a 200-fold excess of λ-DNA. Nucleosome core particles were then separated by native gel electrophoresis in 5% polyacrylamide gels containing 10% glycerol run in 20 mM Hepes pH 8, 1 mM EDTA at 15 mA for 3 h. Gels were dried and nucleosome–protein complexes were visualized by autoradiography.

### 2.5 Surface plasmon resonance (SPR)

SPR binding studies were performed using a Biacore 2000/3000 and T100 (GE Healthcare) at 25 °C in 20 mM Tris pH 7.5, 200 mM NaCl, 0.05% (v/v) polysorbate 20. Biotinylated histone peptides (Millipore) were immobilized onto streptavidin sensor (GE Healthcare) [16]. Different concentrations of CHD4 construct were injected onto the sensor chip (flow rate 100 μl/min to minimize mass transport effects). Experiments were repeated three times. Sensorgrams were corrected for bulk solute and when appropriate, globally fitted to a second-order association reaction. The equilibrium dissociation constants *K*_d_ values were estimated from the concentration dependence of the steady-state response.

### 2.6 ATPase activity assay

The steady-state rate of ATP hydrolysis was determined using the phosphate release assay EnzCheck (Invitrogen) in a standard buffer (40 mM Tris pH 7.5, 50 mM NaCl, 5 mM MgCl_2_) at 30 °C as described in [17].

### 2.7 SAXS data collection and processing

The solution small angle X-ray scattering (SAXS) data were collected using synchrotron radiation at ESRF beamline ID14-3 (Grenoble, France). Ten frames were collected and processed by in-house software. Background subtraction and data quality appraisal were done using PRIMUS program package [18]. Indirect transformation method was used to estimate the maximum dimensions (*D*_max_) in GNOM [19–21]. Ab initio shape reconstruction was done using programs DAMMIN [21] and DAMMIF [22]. Multiple models were averaged (DAMAVER) and filtered (DAMFILT) to the estimated excluded volume [23] and superimposed (SUPCOMB [24,25]). SASREF was used to fit the tPHD, tCHD ab inito models and the ATPase atomic model (PDB: 3MWY [26]) into the tPHDtCHD/ATPase scattering data [27].

## 3 Results

**Supplementary Figure 1:**
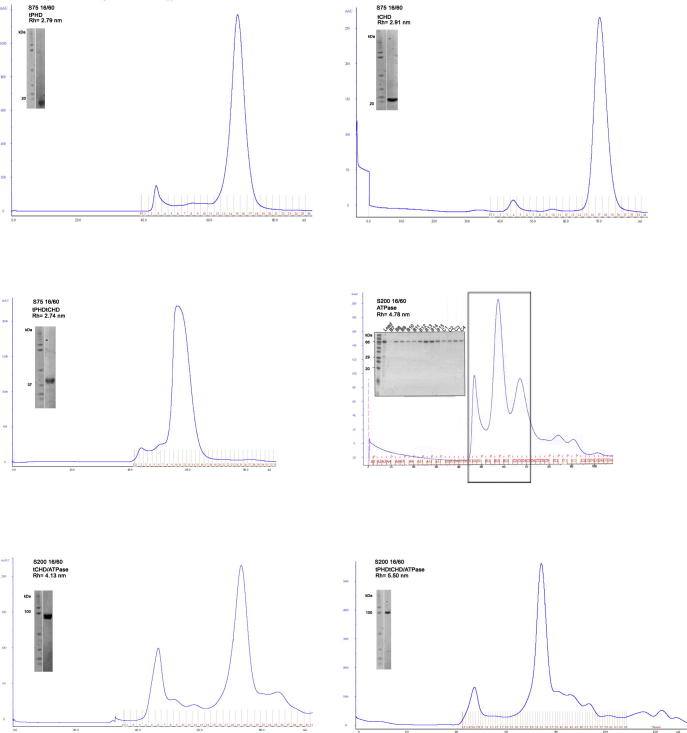
Size-exclusion chromatograms of CHD4 domain constructs. Elution profiles of normalized UV absorbance versus elution volume for size-exclusion chromatography of tPHD, tCHD, tPHDtCHD, ATPase, tCHD/ATPase and tPHDtCHD/ATPase with the corresponding SDS–PAGE gel analysis of either pooled or individual fractions.

**Fig. 1:**
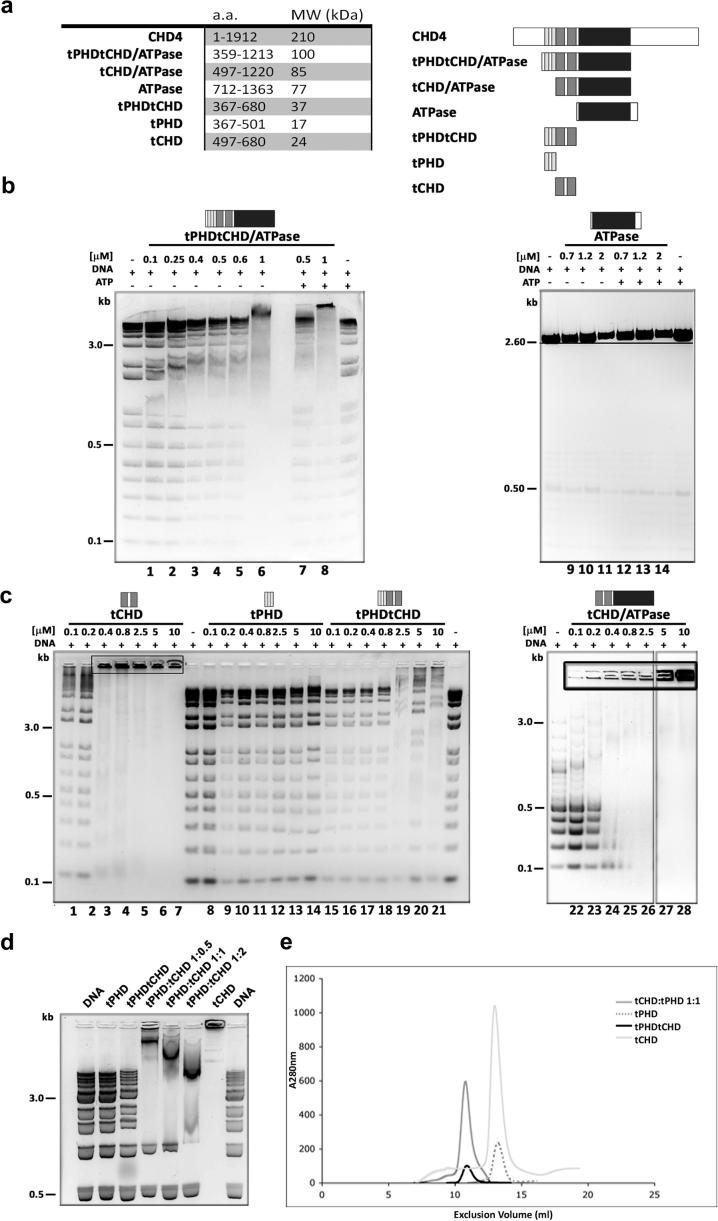
Domain construct design and DNA binding. (a) Schematic representation and molecular weights of recombinant CHD4 domain constructs. Individual domains are color-coded: PHD (light grey), CHD (dark grey), ATPase (black). (b, c and d) EMSA for the CHD4 domain construct DNA-binding activity. The indicated concentrations of recombinant proteins and 1 μg of DNA MW marker were used. Large DNA/protein complexes retained in the loading wells are highlighted inside black boxes. In (d) a mixture of tCHD and tPHD (tCHD:tPHD) at the indicated ratio were used. (e) Gel filtration analysis of tPHD (17 kDa, light gray), tCHD (24 kDa, dotted line), tPHDtCHD (37 kDa, black) and tCHD:tPHD mixture on Superdex G-75 column.

In order to dissect the individual roles of the tPHD, tCHD and ATPase of the human CHD4 chromatin remodelling factor, we created a panel of domain constructs (Fig. 1a). All the proteins were purified to homogeneity and were assessed to be monodisperse by dynamic light scattering (DLS) (Supplementary Fig. 1).

### 3.1 The nucleic acid binding activity of the tCHD is modulated by the adjacent tPHD

We tested whether a minimal construct containing all three domains (tPHDtCHD/ATPase) of human CHD4 is able to bind dsDNA, whether the tCHD is responsible for such binding, and if this interaction is influenced by the adjacent tPHD and ATPase domains. The ability of different constructs to bind dsDNA was probed by mobility shift assays using a DNA ladder as a way of probing binding to differently sized molecules. The tPHDtCHD/ATPase retarded larger (>0.5 kbp) DNA fragments irrespective of ATP (Fig. 1b). In contrast, the ATPase exhibited weak binding, detectable only at high protein concentrations. The gradual increase of the observed DNA probe shift with protein concentration indicates that binding is not sequence specific and that more than one protein may bind to the same DNA fragment.

**Supplementary Figure 2:**
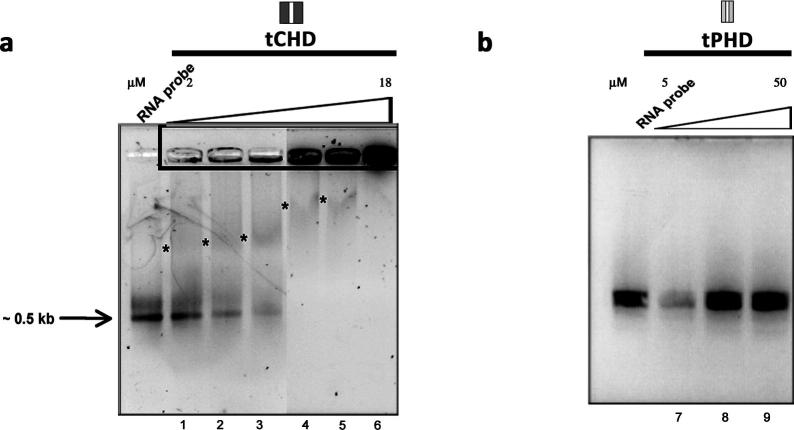
RNA binding studies of CHD4 domain constructs. RNA and protein were incubated together for 5 min, electrophoresed through 2% agarose gels and visualised by ethidium bromide staining. (a) Increasing amounts of tCHD (2–18 pmol) were incubated with a 500 nucleotides ssRNA probe. Large RNA/protein complexes retained in the loading wells are highlighted inside black boxes. RNA/protein complexes are marked by asterisks. (b) Increasing amounts of tPHD (5–50 pmol) were incubated with a 500 nucleotides ssRNA probe.

We next tested the binding affinity of isolated tPHD and tCHD for dsDNA. When incubated with the tCHD, the dsDNA probe was retained in the loading well, forming complexes too large to enter the gel (Fig. 1c) unless very low concentrations were used. The tCHD also exhibited ssDNA (data not shown) and ssRNA binding (Supplementary Fig. 2). In contrast, tPHD failed to bind nucleic acids (Fig. 1c, Supplementary Fig. 2).

A construct, containing both tPHD and tCHD (tPHDtCHD) exhibited dsDNA binding similar to that of tPHDtCHD/ATPase without aggregation (Fig. 1c). In contrast, a construct consisting of the ATPase domain and tCHD (tCHD/ATPase) aggregated in presence of DNA unless very low tCHD/ATPase concentrations were used, i.e. similar to tCHD alone. These results suggest that while the tCHD is sufficient for binding nucleic acids, the tPHD modulates this activity by preventing formation of aggregates**.** Interestingly, aggregation could be prevented by titrating tPHD into tCHD (Fig. 1d) which yields a non-covalent tPHD/tCHD complex (Fig. 1e, Table 1). In conclusion, while the tPHD does not directly bind dsDNA, it closely associates with the neighboring tCHD and affects the mode by which the latter binds dsDNA.

### 3.2 tPHDtCHD moiety binds nucleosome core particles

**Fig. 2:**
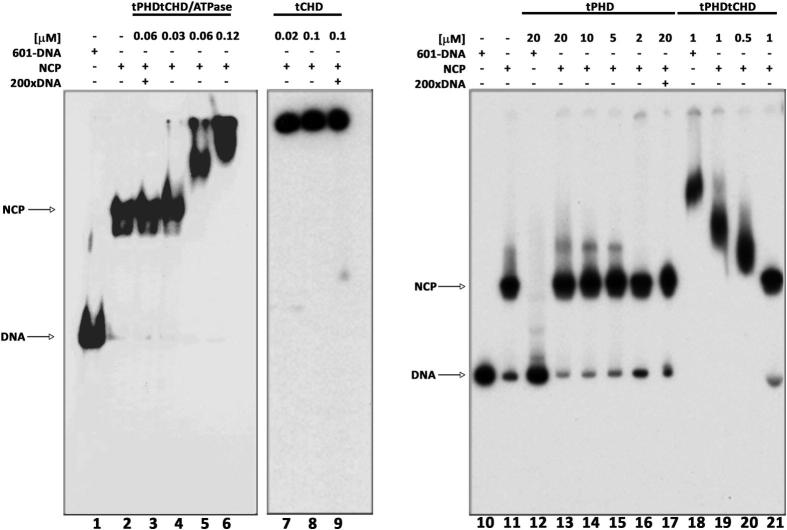
Nucleosome core particle binding to CHD4 constructs. The indicated concentration of recombinant CHD4 constructs were incubated with either 50 fmol of 168 bp-radiolabeled NCPs or with 50 fmol of free 168 bp 601-DNA probe as indicated. The position of NCP and free 168 bp 601-DNA are indicated.

We tested the capability of the various isolated CHD4 domains to bind nucleosome core particles (NCPs). The tPHDtCHD/ATPase caused a significant shift in the mobility of the NCPs (50 fmol) (Fig. 2), the magnitude of which depended upon protein concentration, indicating formation of dynamic complex with moderate affinity. In contrast, upon addition of the tCHD, the NCPs were retained in the loading well, due to formation of large complexes which resisted competition of 200-fold excess of unlabeled DNA. This could be due to formation of very stable tCHD-nucleosomes aggregates or stripping of the nucleosomal DNA followed by aggregation. The isolated tPHD caused only a slight mobility shift of the nucleosome probe, the magnitude of which was independent of protein concentration indicating formation of a low affinity protein–nucleosomes complex of fixed stoichiometry. Finally, tPHDtCHD caused protein concentration-dependent mobility shift, indicating formation of protein–nucleosomes complexes as observed for tPHDtCHD/ATPase. The disappearance of the 168 bp 601-DNA band from the gels in Fig. 2 is consistent with the above demonstrated affinity of tPHDtCHD and tPHDtCHD/ATPase (but not the tPHD) for free DNA. However, the shift seen with nucleosomes is clearly distinct from that observed with DNA alone (c.f. lanes 18 and 19 in Fig. 2).

These results demonstrate that the tPHD domain is necessary and sufficient for nucleosome binding. The tCHD and ATPase domains further increase the binding affinity presumably by binding to DNA that becomes transiently released from the core and/or by allosterically increasing the affinity for histone tails (c.f. lower concentration necessary for binding of tPHDtCHD/ATPase, 0.06 μM and tPHDtCHD, 0.5 μM, versus tPHD, 10–20 μM, Fig. 2). To test if the shifted bands represented intact NCPs and not just protein–DNA complexes, the reactions were repeated under the same conditions, followed by a chase with an excess (200×) of unlabelled dsDNA. If nucleosomes were disrupted then the released nucleosome cores would be competed out by the chase DNA and the NCP band would disappear. The chase DNA effectively removed the band corresponding to the NCP:tPHDtCHD/ATPase complex and restored the band corresponding to intact NCPs. Hence, the nucleosomes remained intact when in the complex with tPHDtCHD/ATPase.

NCPs were reconstituted from recombinant histones lacking post-translational modifications. Binding of tPHD, tPHDtCHD and tPHDtCHD/ATPase to these NCPs suggests that CHD4 is able to recognize unmodified histones, in agreement with previous work [28].

### 3.3 tPHD histone H3 binding affinity is increased by adjacent tCHD

**Fig. 3:**
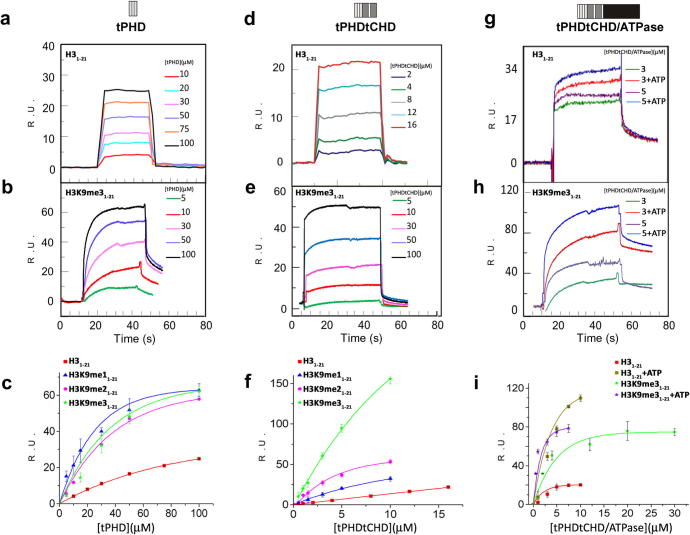
Binding of CHD4 constructs to histone H3 peptides. Binding of recombinant CHD4 domain constructs to immobilized biotinylated histone H3 peptides. (a,b,d,e,g,h): Representative sets of experimental sensorgrams from typical equilibrium-based binding experiments after reference subtraction showing equilibrium response units (RU) as a function of time. Different concentrations of (a, b) tPHD, (d, r) tPHDtCHD and (g, h) tPHDtCHD/ATPase (with and without added ATP) were injected over surfaces coupled with H3_1–21_ or H3K9me3_1–21_ respectively. (c, f, i): Plots of the RU from the experimental sensorgrams of surfaces coupled with H3_1–21_, H3K9me1_1–21_, H3K9me2_1–21_ or H3K9me3_1–21_ as a function of protein concentration for tPHD (c), tPHDtCHD (f), tPHDtCHD/ATPase (i) and corresponding fits to a single site saturation-binding model. The error bars are the standard deviation from the mean of three repeats.

Binding affinities of various constructs for histone H3 peptides were determined by surface plasmon resonance (SPR). The isolated tCHD showed no detectable binding to H3_1–21_, either unmodified or methylated on residue K4 (H3_1–21_K4me1,2 and 3) or K9 (H3_1–21_K9me1,2 and 3) (Table 2). For the isolated tPHD a weak binding with fast dissociation rates was observed to either H3_1–21_ (*K*_d_ = 190 ± 14 μM) or to H3_1–21_K9me3 (*K*_d_ = 70 ± 5 μM) but not to H3_1–21_ methylated on K4 (Table 2, Fig. 3). The construct carrying both tPHD and tCHD exhibited a higher affinity and a faster binding to those peptides (i.e. H3_1–21_: *K*_d_ = 14 ± 3 μM, *k*_on_ = 0.05 μM^−1^s^−1^; H3_1–21_ K9me3: *K*_d_ = 21 ± 2 μM, *k*_on_ = 0.03 μM^−1^s^−1^). The construct with all three domains, tPHDtCHD/ATPase, exhibited the highest affinity for H3_1–21_ (*K*_d_ = 4.1 ± 2 μM), mostly due to a slower dissociation rate. Similar affinities were obtained for the binding to H3_1–21_K9me3 (Table 2). Only a marginally tighter binding was observed in the presence of ATP (*K_d_* = 1.4 ± 1.0 μM) and ATP-γ-S while ADP was not effective (data not shown).

Together, our results indicate that the coupled tPHD and tCHD of CHD4 form a histone interacting moiety, whose binding affinity is augmented by inter-domain interactions with the ATPase domain through which ATP binding and hydrolysis may further modulate the binding.

### 3.4 tPHD and tCHD are required for the DNA-stimulated ATPase activity

**Fig. 4:**
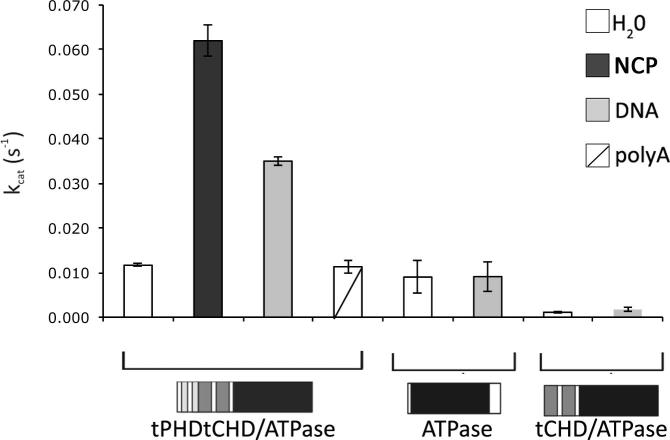
ATPase activity of CHD4 domain constructs. Comparison of the ATPase activity of equal amounts of tPHDtCHD/ATPase, tCHD/ATPase and isolated ATPase recombinant domain constructs. The proteins were incubated with ATP in presence of 0.25 μg of DNA, 0.4 μg of RNA or 0.32 pmol of mono-nucleosome particles (NCP) as indicated. Error bars represent the standard average deviation of at least three independent experiments.

Full-length recombinant CHD4 was shown to possess ATPase activity which is stimulated by nucleosomes and, to a lesser extent, by naked DNA [10,29]. To establish the role of the tPHD and tCHD in the CHD4 DNA-stimulated ATPase activity, we tested and compared the ATPase activity of the various constructs in the presence and absence of DNA. The isolated ATPase domain and tPHDtCHD/ATPase showed weak ATPase activity in the absence of DNA (*k*_cat_ = 0.009 ± 0.0036 and 0.012 ± 0.0003 s^−1^, respectively) as shown in Fig. 4; however addition of DNA significantly stimulated the activity of the tPHDtCHD/ATPase construct (3-fold *k*_cat_ increase at saturating DNA concentration) whereas it had no effect on the activity of the ATPase domain alone. This stimulation was specific for dsDNA since no effect was observed in the presence of RNA (polyA). Interestingly, and in contrast with what was shown previously for dMi-2 [10], the construct tCHD/ATPase showed very weak ATPase activity (*k*_cat_ = 0.001 ± 0.0002 s^−1^), which was not rescued by the addition of DNA and is close to the background. These results suggest that tPHD confers DNA-stimulated ATPase activity by modulating the way in which tCHD binds dsDNA and possibly by interaction with the ATPase domain. The tCHD on its own is not sufficient to confer DNA-stimulated ATPase activity, or perhaps as in yeast CHD1, it may inhibit the ATPase motor [26]. Finally, nucleosomes stimulated tPHDtCHD/ATPase activity about 6-fold (*k*_cat_ = 0.0615 ± 0.0035 s^−1^), comparable to previously reported values [10].

It is interesting to note that tPHDtCHD/ATPase *k*_cat_ values are much lower than those observed for SWI/SNF, ISWI and CHD1. As previously shown the ATPase activity of the full length CHD4 is very low and about one-third that of the native NuRD complex [29], suggesting that other NuRD components may have a role in further assisting CHD4 to achieve optimal ATPase activity.

### 3.5 Low-resolution solution structures reveal domain arrangement

**Supplementary Figure 3:**
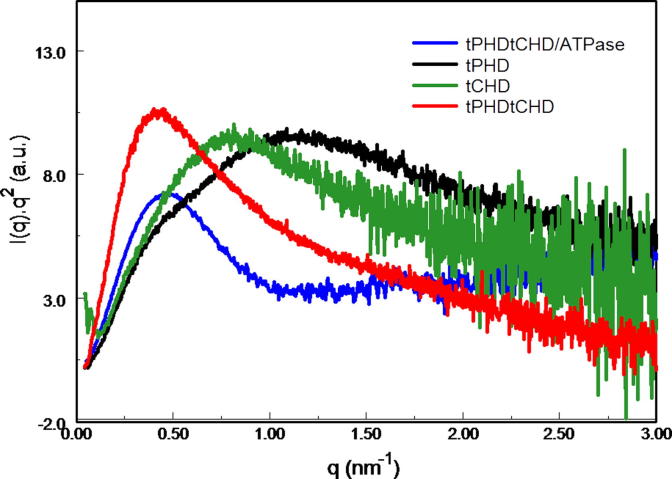
Kratky plots for the CHD4 domain constructs. Kratky plots for the tPHD (black), tCHD (green), tPHDtCHD (red), tPHDtCHD/ATPase (blue) constructs were computed from the averaged and solvent corrected data. The intensities were multiplied to aid visualization.

**Supplementary Figure 4:**
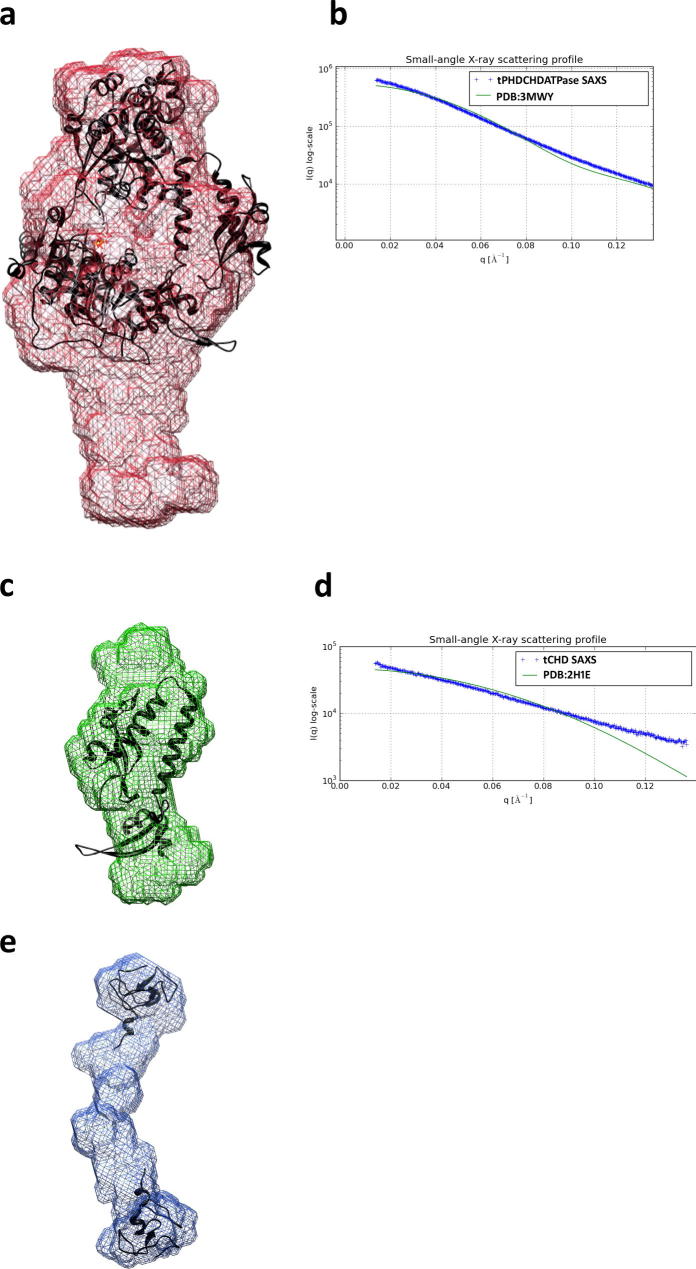
Fit of atomic structures of tPHD, tCHD and ATPase domains into SAXS shapes and calculated versus experimental scattering profiles. Atomic structures were fitted into the low resolution SAXS shapes using the software UCSF Chimera software package (40). Using the same software, calculated scattering profiles based on the atomic structures were calculated and compared to the experimental SAXS scattering profiles. (a and b) Atomic structure of the tandem chromo and ATPase domains of CHD1 (PDB: 3MWY) fitted into the tPHDtCHD/ATPase construct SAXS shape and relative scattering profiles. (c and d) Atomic structure of tandem chromodomains of CHD1 (PDB 2H1E) fitted into the tCHD construct SAXS shape and relative scattering profiles. (e) Atomic structures of the individual PHD1 and PHD2 from CHD4 (PDB: 2L5U and 2L75) fitted into the tPHD construct SAXS shape.

**Fig. 5:**
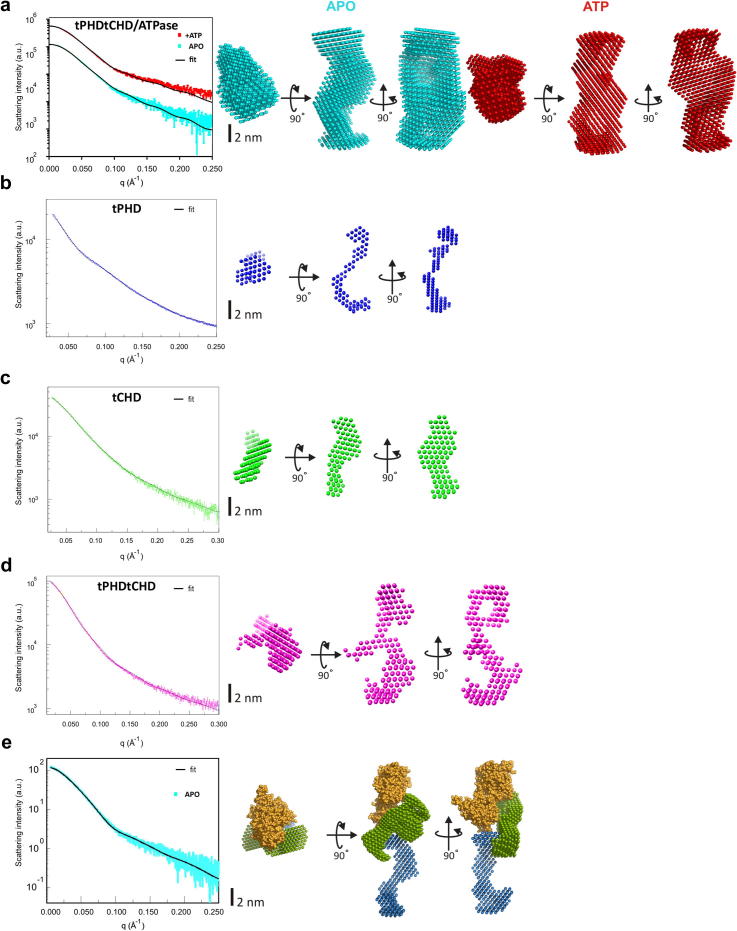
Solution structure and domain modeling. (a–d) Left: experimental SAXS curves and scattering profiles calculated from ab initio models. Right: orthogonal views of average ab initio low-resolution bead models reconstructed by DAMMIF for tPHDtCHD/ATPase (30 models averaged with NSD = 1.008 ± 0.094 (with ATP: red) and NSD = 1.054 ± 0.045 (apo: cyan), respectively), and by DAMMIN for tPHD (blue, 20 models averaged, NSD = 1.123 ± 0.065), tCHD (green, 20 models averaged, NSD = 1.144 ± 0.065) and tPHDtCHD (magenta, 20 models averaged NSD = 1.321 0.093). (e) Domain modeling of the tPHDtCHD/ATPase obtained by SASREF by fitting the tPHD (blue) and tCHD (green) models and the atomic model of the CHD1 ATPase (yellow, PDB: 3MWY) against the solution scattering data of tPHDtCHD/ATPase. Left: Fit of the experimental tPHDtCHD/ATPase SAXS curve (cyan) and scattering profile calculated from the domain model (black line, sqrt(Σχ^2^) = 1.2). Right: Orthogonal views of a bead model representation of the tPHDtCHD/ATPase as obtained by SASREF (tPHD blue, tCHD green, ATPase yellow).

The overall shape and domain arrangement of CHD4 were studied by solution small angle X-ray scattering (SAXS). Scattering curves were checked for concentration dependence and radiation damage and aggregation and only data for monodisperse solutions were included in analysis. Guinier plot parameters were compatible with MALS and DLS analysis (Table 1) indicating asymmetric but folded monomers since Kratky plots [25] exhibited clear maxima (Supplementary Fig. 3). SAXS curves yielded low-resolution models (Fig. 5a–d). The models are consistent with the existing atomic structure individual CHD4-PHD domains (PDB: 2L5U and 2L75 [30]), CHD1 tandem chromodomain (PDB 2H1E [12]) and tandem chromodomain-ATPase (PDB 3MWY [26]) (Supplementary Fig. 4). tPHDtCHD/ATPase scattering curves and models were invariant to ATP binding (Fig. 5a) indicating absence of ATP-driven domain rearrangements.

**Supplementary Figure 5:**
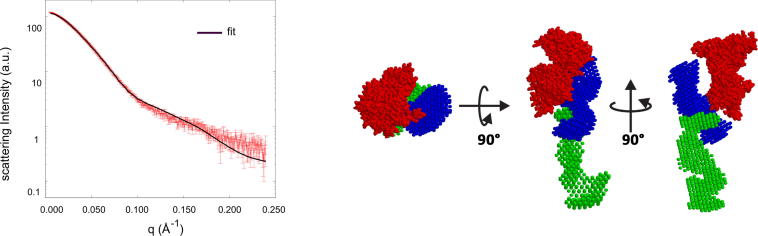
Domain modeling studies of CHD4 domain constructs with SASREF using simultaneous fitting of tPHDtCHD/ATPase and tPHDtCHD SAXS scattering data. Domain modeling of the tPHDtCHD/ATPase obtained by SASREF by fitting of the tPHD (blue) and tCHD (green) *ab initio* DAMMIN/DAMMIF models and an atomic model of the ATPase domain red, obtained from CHD1 crystal structure PDB: 3MWY) simultaneously against the solution scattering data of tPHDtCHD/ATPase and tPHDtCHD. Left: Fit of the experimental tPHDtCHD/ATPase SAXS curve (red) and scattering profile calculated from the domain model (black line, sqrt(Σ*χ*^2^) = 1.2). Right: Orthogonal views of a bead model representation of the tPHDtCHD/ATPase as obtained by SASREF (tPHD blue, tCHD green, ATPase red).

Domain architecture of tPHDtCHD/ATPase (Fig. 5e) was obtained by fitting the atomic structure of the related CHD1 ATPase together with the tPHD and tCHD models (Fig. 5b, c) into tPHDtCHD/ATPase using scattering data constraints. Similar results were obtained by simultaneous fitting against the tPHDtCHD/ATPase and tPHDtCHD data (Supplementary Fig. 5). The resulting model appears slightly more elongated than the tPHDtCHD/ATPase shape (Fig. 5a) since ATPase domain extrudes out from the model, possibly because CHD1 ATPase may not be ideal model for the CHD4 ATPase.

tPHDtCHD/ATPase structure can be divided into two parts: the head and the stalk. The seahorse-shaped tPHD contributes one arm to the stalk domain while the other arm contacts both the tCHD and ATPase within the head domain. tCHD extends towards the stalk contacting tPHD, but shares an extensive interface with the ATPase in the head domain. In effect, the ATPase, which constitutes the bulk of the head domain, is wedged between the arms of both CHD and PHD domains. The intimate association of all domains is fully consistent with the functional interdependence demonstrated above.

## 4 Discussion

Chromatin remodelling ATPases play essential roles in organizing the chromatin landscape which regulates eukaryotic gene expression, yet their mechanism of action is poorly understood. ATP-dependent nucleosome remodelling is likely to be a multi-step process that involves binding of the remodeller to the nucleosome and ATP substrates, ATP hydrolysis, movement of the histone octamer relative to DNA and dissociation of the enzyme from the nucleosome. A tight coordination between these activities is required to achieve efficient remodelling.

The simultaneous presence of tPHD and tCHD is a unique characteristic of the ATP-dependent chromatin remodellers CHD3, CHD4 and CHD5 [14]. However, many chromatin remodelling complexes contain multiple conserved domains which are thought to be involved in chromatin targeting. Although many studies have described the role and properties of these domains in isolation, it remains unclear how these domains influence each other and contribute to the remodelling action and its regulation.

**Supplementary Figure 6:**
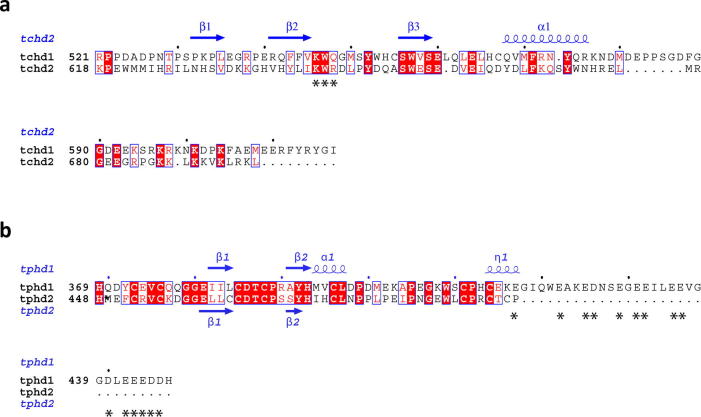
Sequences alignments of individual chromodomains and PHD domains belonging to tPHD and tCHD from CHD4. (a) Sequence alignment of the individual CHD1 and CHD2 domains belonging to the tCHD domain. The secondary-structure elements of the CHD2 atomic structure (PDB: 1MM2) are shown above the aligned sequences. Putative DNA binding sites are marked with a star beneath the sequence alignment. (b) Sequence alignment of the individual PHD1 and PHD2 domains belonging to the tPHD domain. The secondary-structure elements of the PHD1 and PHD2 atomic structures (PDB: 2L5U and 2L75) are shown above and below the aligned sequences. Acidic residues belonging to the 32 amino acid long linker are marked with a star beneath the sequence alignment.

**Fig. 6:**
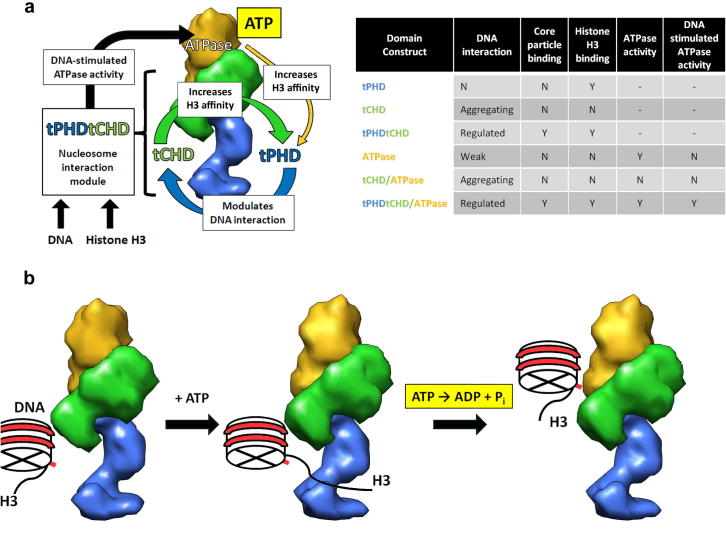
Summary of activities and simple model of ATP hydrolysis cycle. (a) Summary of DNA, core particles and histone H3 binding and ATPase activity of the various CHD4 domain constructs. Nucleosome-recognition by CHD4 is promoted by interdomain interactions within the tPHDtCHD moiety: tPHD modulates DNA binding to tCHD (blue arrow) while tCHD promotes histone H3 recognition by tPHD (green arrow). The tPHDtCHD module allosterically stimulates ATPase when DNA binds (black arrow). The ATPase modulates the H3-tPHDtCHD interaction (yellow arrow). (b) Surface representation of the envelope densities for tPHD (blue), tCHD (green) and the ATPase (yellow) domains. In the absence of ATP, CHD4 binds the nucleosome via interaction between tCHD and fraying nucleosomal DNA. CHD4 binds histone H3 tails through its tCHD/ATPase-regulated tPHD domain. As ATP binds to the ATPase domain affinity for histone tails is further increased. Binding to nucleosomes stimulates the rate of ATP hydrolysis which leads to translocation of the nucleosome substrate along CHD4, straining the contacts between tPHDtCHD and histone tails.

In the present work we show that the two tandem domains of CHD4, tPHD and tCHD, are structurally coupled and modulate each other’s affinity for their respective substrates (Fig. 6a). The tCHD activity is modulated by tPHD domain, which prevents its aggregation into large complexes in presence of DNA. Sequence analysis of tCHD suggests that each individual chromodomain (CHD1 and CHD2) contains a DNA binding motif (Supplementary Fig. 6a). This is consistent with both individual chromodomains of dMi-2 being able to bind to DNA [10]. The presence of two binding sites could effectively crosslink DNA fragments together, thereby creating large aggregates**.** Perhaps, given the physical interaction between tPHD and tCHD domains in the stalk region, tPHD-mediated modulation of tCHD–DNA interaction could be explained by tPHD masking one of the DNA binding sites. Sequence analysis of tPHD shows that a long linker (32 a.a.), which is rich in acidic residues (15 a.a), connects PHD1 to PHD2, making it a plausible electrostatic mask that repels DNA from one of the binding sites on tCHD (Supplementary Fig. 6b).

The tPHD recognizes both un-methylated as well as K9-methylated N-terminal tails of histone H3. The affinity for the H3 tail is increased by the presence of tCHD which does not bind histones. Thus, the effect is most likely structural. The proposed intimate association between tCHD and tPHD domains within the stalk suggests that tCHD may stabilize an active conformation of tPHD and enable it to bind H3 with high affinity (Fig. 6b). Interestingly, the previously measured affinity of the isolated PHD2 domain for the same peptide (*K*_d_ = 18 μM [30]) is very close to the *K*_d_ obtained here for the tPHDtCHD (*K*_d_ = 14–19 μM, Table 2) and is in contrast with the low binding affinity of the tPHD construct in this study. Recently, the isolated PHD1 was also found to bind with similar affinity to H3_1–12_ with *K*_d_ values ranging from 3.2 ± 0.6 μM to 17 ± 5 μM [30,31]. It is possible that the long (32 amino acids) charged and likely flexible linker between PHD domains has an inhibitory effect on the intact tPHD and that only the presence of the tCHD domain restores the correct binding surface.

In our study, we show that the ATPase domain enhances the histone-tail binding affinity of the tPHDtCHD moiety. The ATPase domain greatly increases affinities compared to those observed for the isolated tPHDtCHD (Table 2, Fig. 3). To a lesser degree ATP also modulates the histone tails affinity, a feature potentially important for remodelling. This is consistent with the solution structure studies in which only subtle structural changes may occur during ATP binding.

Vice versa, the tPHDtCHD moiety is essential for the DNA-stimulated ATPase activity since only the tPHDtCHD/ATPase shows DNA-stimulated ATPase activity (Fig. 6). This result could be explained by intimate interaction between the ATPase domain and both tCHD and tPHD within the head part. tPHD arm is wrapped around the ATPase and may stabilize an active configuration or prevent the nearby tCHD domain from sterically blocking access to the ATP or DNA binding sites of the ATPase. This latter possibility is further supported by the observation that the tCHD/ATPase construct, which lacks tPHD, displays severely attenuated ATPase activity that is not stimulated by DNA. These results are in line with the structure of the yeast tCHD/ATPase construct from CHD1 [26], in which the tCHD is seen occluding the DNA binding site of the ATPase motor.

Thus, the intertwined arrangement of DNA binding domains, histone tail binding domains and the motor domain in CHD4 might enable coordination of nucleosome binding and release with the ATP-dependent nucleosome remodelling activity (Fig. 6b). In the ATP-free state, CHD4 is initially targeted to nucleosomes via binding of the tPHDtCHD module to DNA. The tPHD is able to bind H3 tails through recognition of K4 (unmethylated) and K9 (preferably methylated) but the affinities are very low. It is the influence of the tCHD and ATPase domains and the allosteric regulation by ATP binding that increase the tPHD histone H3 tails affinity to biologically significant values. Nucleosome binding stimulates the ATPase activity and hydrolysis (or any subsequent steps) and leads to translocation and change in the relative position of the DNA-bound CHD4 and the nucleosome. The increased strain on the histone binding site may promote dissociation from the tail. This allows mobility of CHD4 between nucleosomes while translocating without compromising the tPHD domain mediated targeting.

These results suggest that the conserved domains flanking the ATPase motor do not only direct CHD4 to the correct substrate but also participate in the remodelling activity. Since this combination of a motor domain and “chromatin targeting” domains is the unifying feature of all chromatin remodellers, we propose that inter-domain allosteric regulation might be a general feature of this class of enzymes.

NOTE ADDED IN PROOF: Since this work was being revised a paper which describes a similar overall shape and domain interactions of CHD4 has been published [32].

## Figures and Tables

**Table 1:** SAXS, DLS and MALS parameters.

		SAXS parameters			DLS	MALS
Protein	MW (kDa)	Excluded volume (nm^3^)	*R*_g_ (nm)	*D*_max_ (nm)	R_h_ (nm)	MW (kDa)
tCHD	24	28	3.06 ± 0.18	10	2.91	25.0 ± 0.8
tPHD	17	20	3.50 ± 0.01	10	2.79	18.0 ± 0.2
tPHDtCHD	37	44	3.73 ± 0.02	11	2.74	39.0 ± 1.1
tPHDtCHD/ATPase	100	122	4.85 ± 0.14	14	5.50	99.0 ± 0.6
tPHD:tCHD	41	–	–	–	–	39.0 ± 1.2

**Table 2:** H3 peptides binding parameters measured by SPR.

Protein	Peptide	*K*_d_ (μM)	*k*_on_ (μM^−1^s^−1^)	*k*_off_ (s^−1^)
tCHD	H3_1–21_	N.D.	–	–
	H3_1–21_K9me1,2,3	N.D.	–	–
	H3_1–21_K4me1,2,3	N.D.	–	–

tPHD	H3_1–21_	190 ± 14 (188 ± 24)⁎	0.0026 ± 0.0002	0.49 ± 0.05
	H3_1–21_K9me1	42 ± 1	–	–
	H3_1–21_K9me2	63 ± 4 (51 ± 5.7)⁎	0.0039 ± 0.0002	0.20 ± 0.02
	H3_1–21_K9me3	70 ± 5 (48 ± 10)⁎	0.0035 ± 0.0004	0.17 ± 0.03
	H3_1–21_K4me1,2,3	N.D.	–	–

tPHDtCHD	H3_1–21_	14 ± 3 (19 ± 3)⁎	0.050 ± 0.010	0.68 ± 0.03
	H3_1–21_K9me1	24 ± 2	–	–
	H3_1–21_K9me2	8.6 ± 5	–	0.31 ± 0.04
	H3_1–21_K9me3	21 ± 2 (10 ± 2)⁎	0.033 ± 0.007	–
	H3_1–21_K4me1,2,3	N.D.	–	–
tPHDtCHD/ATPase	H3_1–21_	4.1 ± 2.0⁎	.0033 ± 0.0021#	0.0135 ± 0.0030
	H3_1–21_K9me1	2.9 ± 9.0 ⁎	0.0009 ± 0.0278#	0.0260 ± 0.0010
	H3_1–21_K9me2	4.7 ± 3.0⁎	0.0012 ± 0.0008#	0.0056 ± 0.0013
	H3_1–21_K9me3	2.6 ± 1.0⁎	0.0010 ± 0.0037#	0.0250 ± 0.0020
	H3_1–21_K4me1,2,3	N.D.	–	–

tPHDtCHD/ATPase+ATP	H3_1–21_	1.4 ± 1.00⁎	0.0189 ± 0.0150#	0.0265 ± 0.0030
	H3_1–21_K9me1	6.9 ± 3.00⁎	–	–
	H3_1–21_K9me2	1.7 ± 2.00⁎	0.0043 ± 0.0053#	0.0073 ± 0.0005
	H3_1–21_K9me3	0.5 ± 0.02⁎	0.0360 ± 0.0246#	0.0180 ± 0.0010
	H3_1–21_K4me1,2,3	N.D.	–	–

N.D: not determinable due to low affinity.

⁎ Estimated from steady state plateau.

# Due to mass transport effect, this on-rate was estimated from *K_d_* and *k*_off_.
